# Editorial: Genetic and epigenetic aspects of non-coding RNAs in physiology and disease

**DOI:** 10.3389/fgene.2022.1078405

**Published:** 2022-12-09

**Authors:** Gabriel Adelman Cipolla, Ticiana D. -J. Farias, Angelica Beate Winter Boldt, Amanda Salviano-Silva

**Affiliations:** ^1^ Department of Genetics, Federal University of Parana, Curitiba, Brazil; ^2^ Department of Biomedical Informatics, School of Medicine, University of Colorado Anschutz Medical Campus, Aurora, CO, United States; ^3^ Department of Neurosurgery, University Medical Center Hamburg-Eppendorf, Hamburg, Germany

**Keywords:** non-coding RNA, miRNA, lncRNA, circRNA, gene expression

The non-coding genomic regions harbor many regulatory sites and genes expressing functional RNAs without protein-coding features, the so-called non-coding RNAs (ncRNAs). Many ncRNAs have important roles in tissue development and homeostasis, and their deregulation has been increasingly associated with pathological conditions. Among the main classes of ncRNAs with regulatory functions, microRNAs (miRNAs) and long non-coding RNAs (lncRNAs) seem to stand out as those most investigated in the pathological context, followed by circular RNAs (circRNAs). Sequence and/or epigenetic status variations have been shown to influence ncRNA gene expression and/or secondary structure, consequently interfering with the expression, availability and/or function of their molecular targets. This Research Topic aimed at stimulating knowledge production on this cutting edge field of investigation, and includes eight original and two review articles exploring the genetic aspects of ncRNAs in physiological states and pathological conditions.

The first article published in this topic reported pioneer information on the levels of ncRNAs in extracellular vesicles (EVs) from anterior pituitary of Duroc swine model obtained by RNA sequencing (RNA-seq) analysis. The authors identified 416 miRNAs, 16,232 lncRNAs, and 495 circRNAs expressed in swine anterior pituitary EVs, predicted signaling pathways and discussed their potential crosstalk with messenger RNAs (mRNAs) as in competing endogenous RNA (ceRNA) networks (Xiong et al.). A second work also focusing on EVs aimed at revealing the profile and predicting the roles of circRNAs in acute ischemic stroke (AIS). To this end, researchers quantified the levels of circRNAs in exosomes obtained from the plasma of individuals after an AIS episode. Compared to controls, they detected differential levels of 198 circRNAs, out of which roughly half were predicted to possess a translational ability and impact focal adhesion, tight junctions, and endocytosis. In addition, a few circRNAs predicted to take part in molecular pathways relevant for AIS were also proposed as potential biomarkers (Yang et al.). In another study with Brazilian patients, EV-miRNAs previously identified as breast cancer biomarkers in serum (miR-320a and miR-4433b-5p) presented high specificity and sensitivity as cell-free miRNAs and in biopsies. This was also true for lower levels of miR-150-5p and higher levels of miR-142-5p in tissue, according to TCGA. In this work, the researchers finally propose combinations of these four miRNAs to distinguish breast cancer patients from controls (Carvalho et al.). Furthermore, differentially expressed miRNAs enriched in leukemia pathogenesis pathways separate acute lymphoblastic leukemia (ALL) from myeloid leukemia (AML) and control individuals. Upregulated miRNA levels in ALL patients was positively correlated with soluble Human Leukocyte Antigen G molecule (sHLA-G) levels, suggesting the potential post-transcriptional regulation of an immuno-regulatory HLA-G and its role in modifying the immune response to subtypes of leukemia (Almeida et al.).

In their review, Ruffo et al. summarized the main results from RNA-seq studies concerning miRNAs, lncRNAs and circRNAs in neurodegenerative diseases, with a special focus on amyotrophic lateral sclerosis (Ruffo et al.). The second review article within this Research Topic addressed the host-virus molecular battlefield. Specifically, the authors presented an overview of the interplay between host miRNAs and HIV molecules or host genes somehow important in the viral cycle. As this remains an underexplored field, they also reviewed the broader literature for miRNAs that regulate, in the context of different diseases, HIV-associated genes. As discussed by the authors, this may be important to exploit microRNA-regulated pathways as potential therapeutic targets against HIV infection (Chinniah et al.). Also on the topic of human viral infections, the pioneering study by Carvalho-Silva et al. revealed the changes in miRNA levels from plasma of patients in the acute and recovery phases of Zika virus (ZIKV) infection. Interestingly, the authors found most differentially quantified miRNAs with lower levels in the ZIKV acute phase in comparison to the recovery phase or to the absence of infection. They highlight miR-142-3p as an example of a miRNA with lower levels in plasma of patients in the acute phase and with important roles in ZIKV infection or in immune responses (Carvalho-Silva et al.). Importantly, human papillomavirus (HPV) infection is recurrent in penile cancer (PeCa) cases and may downregulate two important tumor-suppressor genes *TP53* and *RB1*. Da Silva et al. observed that miRNAs upregulated in PeCa tumor samples potentially interact with *TP53* and *RB1* mRNAs contributing to their low expression. In addition, differentially expressed miRNAs were found to be located in high-risk HPV16 genotype integration sites of the host’s genome, likely influencing the miRNA and target-gene regulation role in cancer pathogenesis (Da Silva et al.). Another cancer with a strong environmental factor is lung cancer. By studying lung squamous cell carcinoma (LUSC) in patients with an undeniable smoking history, Zhang et al. comprehensively analyzed the value of a set of lncRNAs to predict clinical aspects, prognosis, and tumor microenvironment, aiming on optimizing clinical decision making and prompting personalized therapy (Zhang et al.).

Finally, in an RNA-seq analysis of the myocardium tissue of a diabetic cardiomyopathy (DCM) rat model, Xi et al. found 355 lncRNAs and 828 mRNAs to be aberrantly expressed in DCM. Five lncRNAs - including the validated transcript XR_001842089 - were predicted in ceRNA network analysis to have maximum connections with differentially expressed mRNAs, being the AURKB, MELK and CDK1 transcripts their main potential targets in DCM development. According to the authors, these lncRNAs may have clinical relevance for DCM, as they are associated with fibrosis, energy metabolism of cardiac myocytes, and cell proliferation pathways (Xi et al.).

In conclusion, the regulation of gene expression involving ncRNAs in health and disease has a pivotal role in the maintenance of appropriate physiological conditions or sustained inflammation and tumoral processes. Their importance in different diseases cannot be underestimated. However, there is still a long way to firmly establish their causal roles in health and disease, and functional as well as clinical studies are especially needed to confirm their prognostic and diagnostic value. This article collection enables the reader to build new working hypotheses in order to overcome this challenge and achieve a deeper understanding of the physiological/pathological roles of ncRNAs, in addition to their increasing value as accessible biomarkers in different diseases.

